# Development of a Deep Learning Model to Assist With Diagnosis of Hepatocellular Carcinoma

**DOI:** 10.3389/fonc.2021.762733

**Published:** 2021-12-01

**Authors:** Shi Feng, Xiaotian Yu, Wenjie Liang, Xuejie Li, Weixiang Zhong, Wanwan Hu, Han Zhang, Zunlei Feng, Mingli Song, Jing Zhang, Xiuming Zhang

**Affiliations:** ^1^ Department of Pathology, The First Affiliated Hospital, College of Medicine, Zhejiang University, Hangzhou, China; ^2^ Department of Computer Science and Technology, Zhejiang University, Hangzhou, China; ^3^ Department of Radiology, The First Affiliated Hospital, College of Medicine, Zhejiang University, Hangzhou, China

**Keywords:** HCC classification, pathological images, deep learning, whole-slide image, noisy annotation

## Abstract

**Background:**

An accurate pathological diagnosis of hepatocellular carcinoma (HCC), one of the malignant tumors with the highest mortality rate, is time-consuming and heavily reliant on the experience of a pathologist. In this report, we proposed a deep learning model that required minimal noise reduction or manual annotation by an experienced pathologist for HCC diagnosis and classification.

**Methods:**

We collected a whole-slide image of hematoxylin and eosin-stained pathological slides from 592 HCC patients at the First Affiliated Hospital, College of Medicine, Zhejiang University between 2015 and 2020. We propose a noise-specific deep learning model. The model was trained initially with 137 cases cropped into multiple-scaled datasets. Patch screening and dynamic label smoothing strategies are adopted to handle the histopathological liver image with noise annotation from the perspective of input and output. The model was then tested in an independent cohort of 455 cases with comparable tumor types and differentiations.

**Results:**

Exhaustive experiments demonstrated that our two-step method achieved 87.81% pixel-level accuracy and 98.77% slide-level accuracy in the test dataset. Furthermore, the generalization performance of our model was also verified using The Cancer Genome Atlas dataset, which contains 157 HCC pathological slides, and achieved an accuracy of 87.90%.

**Conclusions:**

The noise-specific histopathological classification model of HCC based on deep learning is effective for the dataset with noisy annotation, and it significantly improved the pixel-level accuracy of the regular convolutional neural network (CNN) model. Moreover, the model also has an advantage in detecting well-differentiated HCC and microvascular invasion.

## Introduction

Hepatocellular carcinoma (HCC) is one of the most commonly diagnosed cancers, ranking fifth in incidence rate and second in fatality rate in male individuals worldwide (1). An accurate diagnosis of HCC is essential in the choice of treatment options and the overall survival of patients (2, 3). Some pathological features provide guidance for clinical treatment and prognosis, for example, microvascular invasion (MVI) and microsatellite nodules. However, the current practice of pathological diagnosis of HCC is time-consuming and completely relies on the subjective experience of a pathologist, which varies substantially.

Machine learning, especially deep neural networks, has made appreciable breakthrough in recent years (4–6). The successful application of this technology in medicine includes diabetic retinopathy detection (7), machine learning model-derived predictive gene signature for gastric cancer (8), and artificial intelligence (AI)-based prediction of origins for cancers of unknown primary (9). The transformation of practice from microscope to digital slides has paved the way for using AI assistance systems in pathology. AI-driven approaches for pathological images can assist pathologists in diagnosis and provide clinicians with prognostic stratification and prediction of treatment response. Recent studies have confirmed the effectiveness of pathological AI for tumor detection of various organ systems, such as stomach (10, 11), lung (12), and breast lymph node metastasis (13–15) and prostate core needle biopsies (15, 16). Previous studies of HCC based on convolutional neural network (CNN) mainly focused on radiology images, including CT scans, ultrasound, and MRI scans (17). Li et al. (18) proposed a structure convolutional extreme learning machine and case-based shape template methods for HCC nucleus segmentation. However, various tissue structure and cell characteristics should be comprehensively considered for the diagnosis and histological grade of HCC. There are few AI-based studies on large sample whole-slide images (WSI) of HCC.

AI-based pathology usually requires a large number of accurate annotations, while the manual annotating of histopathologic images is very time-consuming and hard to be precise. Furthermore, there exists a problem that the larger the scale we use to get more complete cellular features, the higher probability of selecting noisy patches—for instance, a larger annotated tumor region may contain fibrous stroma and blood vessels. In addition, sporadic cancer cells around the primary tumor are difficult to be fully marked. These conditions make annotations of pathological digital slides scarce and noisy, both of which are detrimental to machine learning models. Song et al. (19) add artificial noisy images as negative samples to simulate the influence of blood vessels and stains, but the artificial images are oversimplified and cannot fundamentally change the original annotations. It brings us to the crucial problem in AI-driven diagnosis for HCC pathological images, which is noisy annotation.

In this study, we conducted a series of experiments on slide screening and scale selection to solve the noise problem. Furthermore, we introduced label smoothing to implement soft constraints. Compared to other methods that directly use the noisy label to optimize the model, our method can automatically eliminate the influence of incorrect labels instead of being misguided by them. The independent validation set demonstrated that, through patch screening and noise-tolerant loss function, our proposed method showed better results than the traditional data processing approach on the noisy dataset.

## Materials and Methods

### Patients and Samples

A total of 592 HCC patients treated by surgical resection at The First Affiliated Hospital, College of Medicine, Zhejiang University between 2015 and 2020 were enrolled in the study. Patients who had undergone prior radiotherapy or chemotherapy and combined with intrahepatic cholangiocarcinoma or mixed hepatocellular–cholangiocarcinoma were excluded. We collected an HCC pathological image dataset containing hematoxylin and eosin (H&E)-stained digital slides from 592 patients with diverse HCC subtypes and differentiation degrees. The digital slides were produced at ×40 magnification by the 3DHISTECH P250 FLASH digital scanner.

### Data Set Preparation

One representative H&E-stained digital slide was available for each HCC case, which included HCC tissues and the adjacent surrounding liver tissues. An expert liver pathologist made the rough annotations of the tumor regions, including 20 slides which were elaborately annotated for pixel-level evaluation. Among all the samples, more than 400 cases belong to grade 2 or grade 3 based on the Edmondson–Steiner grading system. Concerning the imbalanced slide amount of tumor differentiation degree, we made a preliminary filtrating to 137 cases for training, and the remaining 455 slides were used for validation and testing (211 slides of the validation set and 244 slides of the testing set). Besides this, we also collected 157 slides in The Cancer Genome Atlas (TCGA) database as an external testing set for testing the robustness of our model. It is worth noting that our task is to detect the HCC region, so all the other tissues, including liver cirrhosis, are labeled as grade 0.

To get the multi-scaled patch, hundreds of central points with a minimum distance were selected on each slide. For each point, three patches were cropped at ×5, ×20, and ×50 magnification to retain the information of different scales. We resized all of them to 448 pixels to get a set of patch groups with the same size and position. The label of each patch was determined by its relative position to the annotated tumor region. For the training set, a certain number of patches were randomly chosen in each slide. It is worth noting that these patches were used for pre-training the model. As for the validating set, we artificially selected a small number of patches with correct labels to obtain an accurate assessment. All these patches are pre-processed by stain normalization before training.

### Data Screening

**Figure 1:**
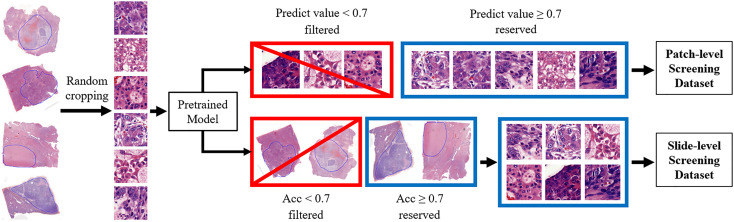
Illustration of the process of our proposed method. Firstly, all slides of the training set were cropped randomly and used for model pretraining. Secondly, the pretrained model is trained with about 85% accuracy and then used for two different dataset screening methods. For patch-level screening, all patches were independently assessed by patch-level screening and would be filtered if the predicted value was below 0.7. This threshold is determined by the ablation study. For slide-level screening, patches cropped from the same slide are calculated together to filter the slides with low quality. Only patches of slides with 70% overall accuracy would be retained, which means that the final dataset would not contain slides with unreliable annotation.

The above-mentioned training set was used to train preliminary models for screening, which can obtain the model with an accuracy of about 85% on the validating set. Since we used data with rough annotations, there existed some slides with high noise. In these slides, it is complicated for the pathologist to annotate accurately on all these small regions. Considering that the slide size is hundreds of times larger than the cropped patches, it is impractical to ensure the accuracy of the patch-level labels, resulting in the dataset with noisy labels. One of our strategies for handling noisy data was slide-level screening, which means to retest all the slides severally by pretrained models and eliminate the patches of slides with low accuracy. Differently, the patch-level screening would directly test on patches and eliminate the incorrectly predicted ones. The pre-trained model was not highly accurate, so ambiguous patches would be retained in the screened dataset. It means that patch-level screening reduces the dataset noise, but the remaining noisy patches might be highly mistakable. The slide-level screening focuses on the accuracy of each slide instead of the patch, so noisy patches are filtered without strains of complexity. Both screening methods have their pros and cons in different conditions. As per the process shown in **Figure 1**, in the case that the model has 85% accuracy, low slide-level accuracy of less than 70% demonstrates that the slide contains lots of labeling errors. These noisy samples can obstruct effective feature extraction during training and need to be removed. For patch-level screening, we filtered patches directly with predicted values of less than 0.7. Then, two different training sets were prepared for the next stage. Here the threshold is determined by the ablation study. The models were trained on different datasets with a threshold of 0.6, 0.7, and 0.8 to get accuracies of 91.58% (0.009), 93.03% (0.008), and 92.12% (0.010), respectively.

In consideration of the data imbalance problem, we adjusted the patch quantity of different categories and different patients, which avoided the model from having a preference. The training samples were selected from filtered data, totaling 22,800 patches from 137 slides. The validating set contained 412 patches of 211 slides by manual screening. Only a few patches of the same slide were selected. In increasing the diversity of the dataset, involving more slides was more helpful than more patches of the same slide. Unlike these processing methods, each sample of the testing set retained whole-slide images to evaluate the diagnosis results with slide-level accuracy.

### Model Design

It usually takes tens or even hundreds of thousands of samples to train an effective model in deep learning. Since it is unrealistic to acquire large amounts of medical samples, we chose to use the model already trained on the other large dataset [ImageNet dataset (20)]. The pre-trained model would have a feature extraction ability for basic image features. ResNet18 was adopted as the basic architecture of our model. Although the screening methods mentioned above have a certain efficiency in filtration, the screened dataset will still be noisy. Focusing on the abovementioned problem, we also applied a new strategy for noisy HCC annotations based on the CNN model to improve the HCC classification performance.

**Figure 2:**
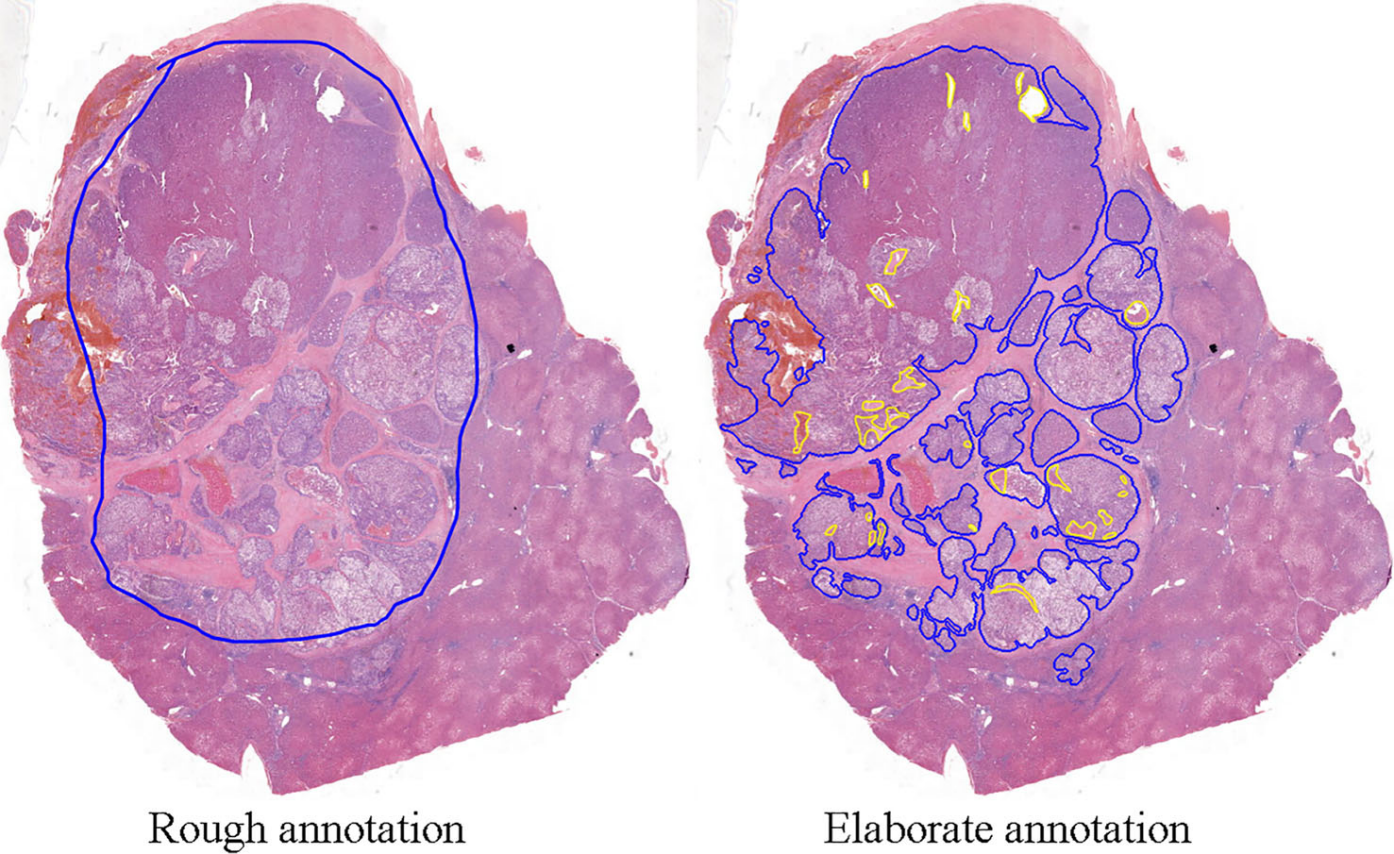
The process of our proposed model. Patches of three scales (×5, ×20, ×50 magnification) were cropped from slides, and the corresponding labels were decided according to annotations. Patches of ×50 magnification are at a large scale containing the clearest cellular features but are also probably noisier. In contrast, ×5 magnification minimizes errors, but the cellular features can be hardly seen. These patches are severally inputted into the main encoder ***E_m_*** and classifier to get results. In the other part, the regular labels are transferred into smooth labels to guide the training in our model. Additionally, our model contains a module to get dynamic smoothing weight by the surrounding information of each patch. Here ***E_p_*** denotes the pretrained Autoencoder to generate the feature polymer, which is utilized to output the dynamic smoothing weight ***ϵ***.

Considering the distinctive characteristics of pathological slides, we took image scale as one of the most significant factors in cancer recognition. In macro-view, the probability of noisy samples will be lower, while the morphology of cells is clearer in micro-view. It means that a proper scale would be crucial in computational HCC diagnosis. In this work, we attempted to examine model performance with patches of various scales and get the proper magnification between features and noise so that enough features were contained in patches and the rate of noisy labels would not be too high. Our dataset contained the patch groups with the same size and different magnifications of ×5, ×20, and ×50. The label of each group was decided by the annotation region. These patches of three magnifications were severally inputted into three models with the same architecture during training for comparison. The process of our proposed model is shown in **Figure 2**.

As for the loss function, we introduced label smoothing into our model for alleviating the influence of residual noisy labels. With cross-entropy loss $ℒ=-\sum_{i=1}^{N}y_{i}\text{log}\left(p_{i}\right)+\left(1-y_{i}\right)\text{log}\left(1-p_{i}\right)$ , the predicted probability of each sample $p_{i}=1/\left(1+e^{-z_{i}}\right)$ will be forced to infinitely approximate to the probability interval [0,1] according to the label. Here *N* denotes the batch size, *y* denotes the patch label, and *p* denotes the predicted value. The latter formula is the sigmoid function mapping model output *z_i_* into the probability interval [0,1] as final probability *p_i_*. This loss function will guide the output *z_i_* toward polarization during gradient descent, resulting in the over-confidence of each sample and consequently reducing model generalization. Differently, smooth label is defined as $\tilde{\text{y}}$ _i_ = (1 - *ϵ*)*y_i_* + *ϵ*/2 where *ϵ* is the hyperparameter that determines the degree of smoothness. With the constrain of smooth labels, the outputs of the model (before the sigmoid layer) will be limited in a fixed range. Rafael et al. (21) found that ordinary labels lead the outputs dispersing into broad clusters through visualization of the activation of the penultimate layer. In contrast, the clusters produced by smooth labels are tighter. The work of Lukasik et al. (22) also demonstrated the superiority of label smoothing for deep learning with noisy labels. In our work, noise exists between two categories. After data screening limits the noise to a lower degree, label smoothing has the ability to restrict the over-fitting caused by noisy labels so that the model generalization can be enhanced.

Differently from (22), the degree of label smoothing should be determined by the surroundings of each patch, so an additional module is added in our model to dynamically choose the smoothing weight. As shown in **Figure 2**, the surrounding patches are formed into the feature polymer, representing the large-scale view without losing cellular information. Here the encoder *E_p_* is pre-trained by Autoencoder. Then, a group of fully connected layers is trained to output the value of *ϵ*. The core idea is that the surroundings of each patch are related to the probability of the label being mislabeled. If the patch is surrounded by a mixture of normal and cancerous cells, the annotated label is not credible, and a large value of *ϵ* should be applied, According to the ablation study of smoothing weight, the new smoothing weight is set as *ϵ*′ = 0.2*ϵ*, where *ϵ* is the output of the sigmoid function between 0 and 1.

### Ethics Statement

This study was approved by the Ethics Committee of The First Affiliated Hospital, College of Medicine, Zhejiang University. Written informed consent was obtained from all patients in this study.

## Results

In the experiments, ResNet18 (23) is adopted as the encoder of the model, which is pre-trained on ImageNet datasets (20). The encoder of the feature polymer consists of six convolutional layers, followed by two fully connected layers to output the weight ϵ. In model training, Adam is used as the optimizer. The batch size is 32, and the learning rate is 0.0001 in all the experiments for fair comparison.

### Slide Screening

To demonstrate the necessity of screening slides to filter out those with high noise, we compared the performance of the same CNN model training on the raw dataset, slide-level screened dataset, and patch-level screened dataset. Furthermore, for each dataset, three magnifications were employed to generate patches. The result is shown in **Table 1**, which demonstrates the incredible advantage of the patch-level screening method in all scales. The accuracy on the patch-level screened dataset is on average 5.10% better than the slide-level screened dataset and 8.92% better than the raw dataset. We got the highest accuracy of 93.03% in the patch-level screening dataset with ×20 magnification. This result illustrates that, with patch-level screening, the ×20 magnification dataset balances the influence of the image noise and the cellular features so that the model achieves high performance in this dataset.

**Table 1:** Comparison of convolutional neural network model performance on multiple-scale datasets with different magnifications.

Scale	Data		
	Raw data	Slide-level screening	Patch-level screening
ACC (***×*** 5)	81.29% (0.017)	86.99% (0.011)	90.85% (0.008)
ACC (***×*** 20)	83.15% (0.022)	86.00% (0.013)	93.03% (0.008)
ACC (***×*** 50)	84.49% (0.021)	87.40% (0.006)	91.80% (0.008)

The variance of 10 trials is shown after each accuracy score. Acc denotes the patch-level accuracy.

### Label Smoothing

After determining the screening method and patch scale, label smoothing was applied to further mitigate the negative effects of the noisy samples. Here we adjusted the hyper-parameter *ϵ* from 0.1 to 0.3 and calculated the patch-level accuracy on the validating set. The results are shown in **Table 2**. According to the results, the weight of 0.2 was adopted in label smoothing. Moreover, to dynamically choose the smoothing weight ϵ, we introduced the feature polymer to integrate surrounding information, and the results demonstrate the significance of our model.

**Table 2:** Patch-level accuracy with label smoothing of different weight ϵ, which was trained on the screened dataset of ×20 magnification and evaluated on a validating set.

Model	Patch-level accuracy			Dynamic *ϵ*
	*ϵ* = 0.1	*ϵ* = 0.2	*ϵ* = 0.3	
Acc	92.17% (0.008)	93.01% (0.004)	92.55% (0.004)	93.87% (0.005)
AUC	0.9572 (0.007)	0.9691 (0.007)	0.9604 (0.011)	0.9720 (0.008)
F1-score	0.9364 (0.016)	0.9585 (0.010)	0.9397 (0.010)	0.9644 (0.012)

The variance of 10 trials is shown after each accuracy score. Acc denotes the patch-level accuracy. AUC denotes the area underneath the entire ROC curve. “F1-score” is the harmonic mean of the precision, and recall that F_1_ = 2 (precision recall) / (precision + recall).

### Slide-Level Accuracy

In slide prediction, all patches of each slide were input into the trained model to get the predicted categories, and the ratio of the cancer patch will be calculated to decide the slide-level prediction according to the threshold. We collected 244 slides in our dataset as an internal testing set, including 161 HCC slides and 83 paracancerous liver tissue slides. Given the necessity of data balance, the slide numbers of all grades according to the Edmondson–Steiner grading system were controlled to be as equal as possible. We calculated the accuracy separately to have an intuitional understanding of the results. In slide prediction, all patches of each slide are input into the trained model to get the predicted categories, and the cancerous ratio of the whole slide will be calculated to decide the slide-level prediction according to the threshold. The results showed that 0.04 was the optimal threshold value for both models, in which the baseline model achieved the accuracy of 97.54% and our model achieved the accuracy of 98.77%. The predicted results and the sample amounts of each grade are shown in **Table 3**. It can be seen that the model accuracy on grade 2 and grade 4 is the same; it might be due to the high similarity of the slides in our dataset. Nevertheless, our model achieves better performance with 98.77% overall accuracy, which demonstrates the superiority of our model.

**Table 3:** Slide-level accuracy results of different grades in internal testing set.

						Total
Baseline model						
Grade	0	1	2	3	4	/
Acc	97.59%(81/83)	93.94%(31/33)	97.92%(47/48)	97.92%(47/48)	100%(32/32)	97.54%(238/244)
Our model						
Grade	0	1	2	3	4	/
Acc	98.80%(82/83)	96.94%(32/33)	97.92%(47/48)	100%(48/48)	100%(32/32)	98.77%(241/244)

We utilized our testing set to determine the optimal threshold of 0.04. The sample amount of each grade is shown after each accuracy. Here grade 0 denotes non-tumor, and the others are Edmondson–Steiner grades. Acc denotes the slide-level accuracy.

**Figure 3:**
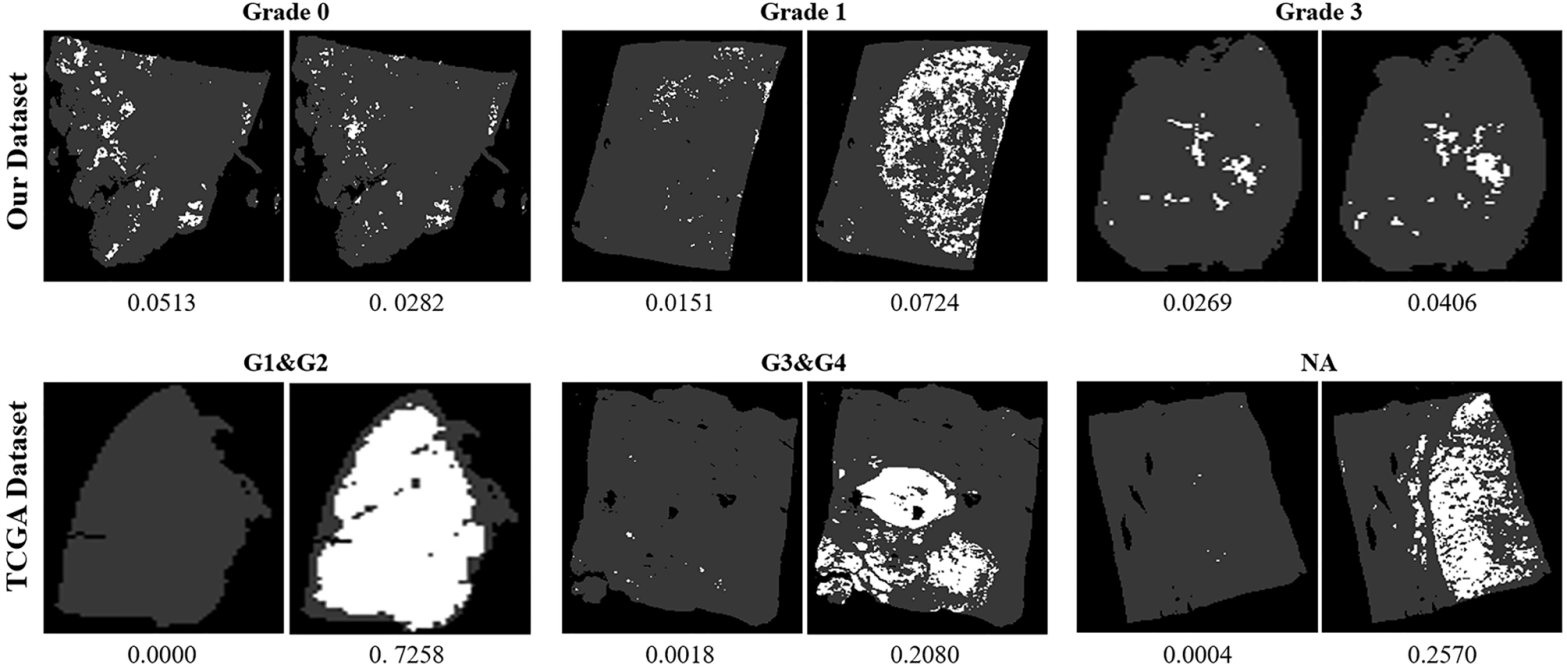
Visualization of slides with different predictions by the baseline and our model. For each slide, the figure to the left is the prediction of the baseline model, and the figure to the right is the prediction of our model. The ratio below each figure denotes the overall ratio of cancerous patches. Except for the slide of grade 0 in our dataset, all the other slides contain tumors with different grades. It can be seen that, although there is not much difference in the predictions of our dataset, the performance of our model is more significant in the public dataset The Cancer Genome Atlas, which reflects the high generalization of our model.

We also collected 157 HCC slides in TCGA database as an external testing set for testing the robustness of our model. Because of the difference of the image acquisition equipment, the models would tend to be less accurate. It is noticeable that our model holds a significant lead over the baseline model with a slide-level accuracy of 87.90%, while the regularly trained CNN model only makes accurate predictions on 54.14% of the TCGA slides (**Table 4**). From the results described above, we conclude that, although the baseline model achieves accuracy close to our model with a specific threshold, it cannott generalize well to other samples like the slides from TCGA. The primary cause of that is that our model uses label smoothing to alleviate the over-fitting problem, resulting in a better ability in recognizing easily confused samples. The slide-level accuracy reflects the superiority of our model in the classification. It is an overall criterion without reflecting details of the prediction. Moreover, the slides with different predictions by the baseline and our model are shown in **Figure 3**. It can be found that the difference between the predictions on our dataset is not obvious, but the performance of our model is more significant in the public dataset TCGA, which means that our model has a high generalization on the different datasets without training. The next step of evaluation is to pay attention to the model prediction on each slide, which helps us to understand why our model performance is better than the regular CNN model.

**Table 4:** Slide-level accuracy results of different grades in The Cancer Genome Atlas database.

				Total
Baseline model				
Grade	G1&G2	G3&G4	NA	/
Acc	47.48%(47/99)	68.09%(32/47)	54.54%(6/11)	54.14%(85/157)
Our model				
Grade	G1&G2	G3&G4	NA	/
Acc	83.84%(84/99)	93.62%(44/47)	90.91%(10/11)	87.90(138/157)

We utilized our testing set to determine the optimal threshold of 0.04. The sample amount of each degree is shown after each accuracy score. Acc denotes the slide-level accuracy.

NA denotes the group of samples without the exact grade.

### Visualization Result

**Figure 4:**
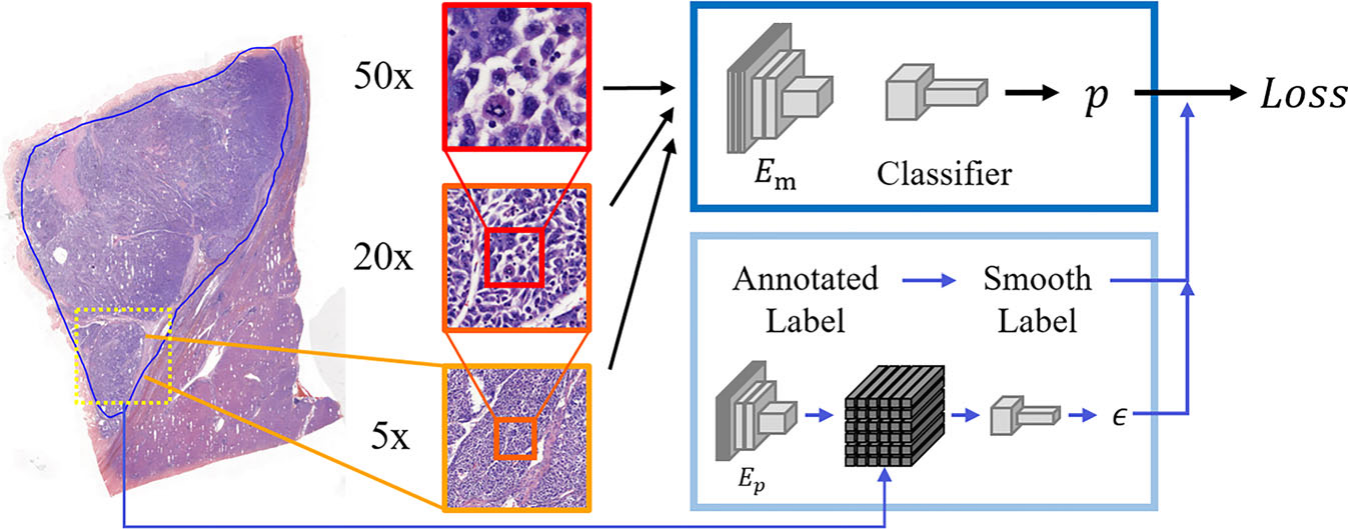
One of the whole-slide images of hepatocellular carcinoma tissues. The width of the image is around 10k pixels. In the figure to the left, the blue line drawn by pathologists points out the tumor regions roughly, containing non-tumor areas without tumor features. In the figure to the right, with elaborate annotation, in addition to the blue line showing the cancer region precisely, the yellow line circles the non-tumor parts within the tumor.

For further assessing the effects of two models on the diagnosis of HCC, we visualized the model prediction on whole-slide images to acquire more intuitive results. A total of 20 independent slides were elaborately annotated for pixel-level evaluation. Compared to the elaborate annotation, the rough annotation always contains some non-tumor components and ignores scattered HCC cells around the major tumor region. One example is shown in **Figure 4**.

Since pathological images are too large for segmentation, the common approach is to utilize the sliding window to calculate the mean prediction of each pixel as the pixel-level accuracy. Since blank areas around the valid region do not contain cells, the model can easily distinguish the patches cropped from these areas. The high proportion of these areas will also highly make the results false. We excluded the blank areas around and within the organization before calculating the accuracy. All the visualization results of precisely annotated slides can be found in **Supplementary Figure S1**. Among all the 20 slides with precise annotation, our proposed model achieves an accuracy of 89.98%, which is higher than the performance of the baseline model of 82.52% and label smoothing of 87.72%.

**Figure 5:**
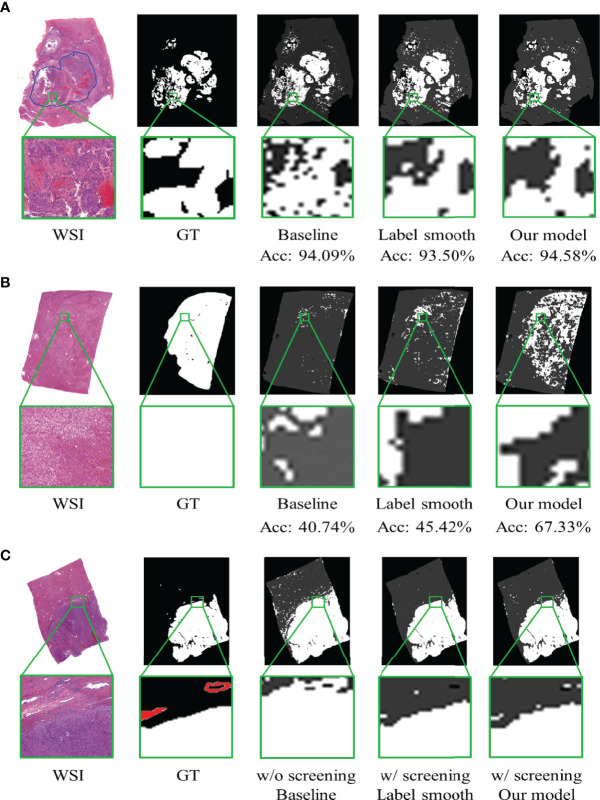
The visualization of model prediction on whole-slide images. Acc denotes the overall accuracy of the slide. **(A)** Differentiation of the proposed model in the suspected area of tumor.** (B)** Recognition of the proposed model in well-differentiated hepatocellular carcinoma. **(C)** Recognition of the proposed model in microvascular invasion (MVI). The white region denotes the tumor, and the red regions denote the MVI.

In addition, the visualization results show that our proposed model has superior ability in differentiating regions mixed with cancer cells and benign cells, recognizing well-differentiated HCC and MVI. As shown in **Figure 5**, the figures from the left to the right represent the whole-slide image, annotated ground truth, predicted map of the baseline model, and our model. Each pixel in the predicted figures is the output of the corresponding patch, and we magnify the same part of the region to have a distinct view. One of the visualization results shown in **Figure 5A** is a complex sample with highly dispersed cancer cells. Significantly, the selected region is located in the cancer region according to the rough annotation, but from the elaborate annotation, we find fibroblasts (left of the region) and erythrocytes in the vessels (right of the region). From the corresponding area of predicted maps, we can see that the baseline model recognizes the erythrocyte area but puzzles at the fibroblasts area on the left. On the contrary, these patches are accurately discriminated by our proposed model. The visualization results also show that our proposed model could distinguish well-differentiated HCC. As shown in **Figure 5B**, the baseline model ignores almost all the well-differentiated cancer cells, while our model accurately predicts most of them. Notably, our proposed model can also help pathologists to identify MVI and microsatellite nodules. Here we show an example in **Figure 5C**, which contains multiple MVI samples (the annotated regions outside the tumor with red circles). From the visualization results, it is clear that the model trained by our screening dataset can recognize several distinguishable MVI samples, avoiding the omission of important information by human diagnosis.

## Discussion

There have been plenty of innovative research on traditional machine learning methods, like support vector machine, random forest, and boosting algorithms (24). In general, the above-mentioned methods have achieved applaudable performance in CT and ultrasound image analysis, which provides a non-invasive and low-cost way for the auxiliary diagnosis of disease. Liao et al. (25) extracted image features from HCC pathological images and then adopted random forest to diagnose HCC and predict survival outcomes. Fehr et al. (26) applied recursive feature selection support vector machine in the binary classification task of prostate cancer. Although traditional machine learning methods have been well applied in these studies, all the approaches are highly dependent on the features extracted by manual rules, which is time- and labor-consuming. The hand-designed features are not likely to fit the data perfectly, leading to the limitation in model performance.

With the popularization of deep learning, various structures of CNN were widely applied in the diagnosis of HCC. Schmauch et al. (27) proposed a deep learning model to differentiate space-occupying lesions in the liver using data of 367 ultrasound images with a classification accuracy of 91.6%. Vivanti et al. (28) proposed an automated detection model based on the CT images of HCC which can identify tumor recurrence with an accuracy of 86%. Hamm et al. (29) described an MRI liver lesion classification system based on CNN with an accuracy of 92%, a sensitivity of 92%, and a specificity of 98%. As the gold standard in medical diagnosis, pathological slides contain more features for conclusive analysis. Indeed the high complexity of slide annotation and gigapixel image processing makes the computer-assisted diagnosis on pathological slides meaningful but also challenging (30).

Unlike other pattern recognition tasks, pathological image diagnosis faces two primary challenges: large image size and noisy annotation (31). Commonly, the size of a pathological digital slide is more than 100,000 pixels, which is too large for an input of a normal model. Simply resizing the whole slide into a small image will make nuclear morphology indistinguishable, leading to the loss of crucial classification information. Most of the research split the whole slide into patches for individual classification and assembled the results back to the original slide. Kiani et al. (32) created a model based on CNN to differentiate HCC from cholangiocarcinoma. They used a total of 25,000 non-overlapping image patches of size 512 × 512 pixels to train the model and yielded an accuracy of 84.2% on an independent test set. Liao et al. (33) designed a CNN model to identify liver tumor tissue from normal. The CNN model was trained and tested on 256 × 256 pixels and yielded an accuracy of 94.9% at ×5 magnification and 86.0% at ×20 magnification, respectively. These methods ignored the macro-view structural information. Besides that, the size problem generally caused the noisy annotation in HCC pathological slides. These noisy annotations will potentially limit the performance of the model. Our proposed method aims to solve these problems during data processing and model training. From the dataset with rough annotation, our method trained with patch-level screening dataset achieved the highest accuracy with 93.03% on ×20 magnification, while regular CNN model with the same architecture only reaches 84.49% accuracy at most. The most significant difference from the above-mentioned research is that our proposed method was devised for classification with noise annotation. Since some components, like fibrous stroma located in the tumor region, are more likely to be annotated as cancer samples, they appended artificial noisy images to the dataset to enhance the recognition capability of the model, but for the preexisting fibrous stroma images, this approach cannot correct the inaccuracy annotations. Moreover, the correction effect largely depended on the quality of the artificial images, so simple artificial images with singular colors were more likely to be distinguished from real noisy images. It is necessary to alleviate the impact of noisy annotations by considering the causes of the problem, such as the rough annotation and the over-fitting of noisy labels.

In practice, our proposed model may assist pathologists in HCC diagnosis. The pathological diagnosis of hepatic atypical hyperplastic nodule and well-differentiated HCC is difficult. Sometimes pathologists need to rely on immunohistochemistry and reticular fiber staining for diagnosis. The cancer features of these slides are not clear enough to be distinguished, leading to neglect of the underlying tumor in the baseline model. Our model can identify early HCC ignored by the baseline CNN model, which is particularly significant in practical diagnosis. In addition, our proposed model can also help pathologists to identify MVI and microsatellite nodules (34). It is well known that MVI and microsatellite nodules are two crucial prognostic indicators of HCC. Song et al. (35) and Wei et al. (36) used deep learning to predict MVI in HCC based on MRI with an accuracy of 79.3 and 81.2%, respectively. However, until now, there is no previous study based on deep learning to enable the diagnosis of MVI. The recognition of MVI is very challenging since most of the MVI samples are too small and consist of few distinguishing features. It is worth noting that our model was not trained by any MVI samples, which highly increased the difficulty of this task. From the visualization of prediction, the model trained on the dataset without screening misclassifies lots of normal cells, but the model trained by our screening dataset not only accurately predicts two types of cells but also recognizes several distinguishable MVI samples, which will be meaningful for diagnosis. Such good results benefit from screening by a pretrained model.

Increasing studies have uncovered that the histological phenotypes of HCC are closely related to gene mutations and molecular tumor subgroups (37)—for instance, CTNNB1-mutated HCCs display a particular phenotype, exhibiting microtrabecular and pseudoglandular architectural patterns (38). Macrotrabecular-massive HCC frequently harbors TP53 mutations and/or FGF19 amplifications, which exhibits a very aggressive phenotype (38, 39). Recently, CNN has been shown to be able to predict genetic alterations and the overall survival of patients with lung and brain tumors, respectively. Coudray et al. (12) found that CNNs can be trained to predict the six frequently mutated genes of lung adenocarcinomas using WSI. The prediction accuracy rate is in between 73.3 and 85.6%. The study conducted by Mobadersany et al. (40) showed that survival CNN models can detect histologic differences associated with isocitrate dehydrogenase mutations in astrocytomas. The establishment of a classification of HCC that integrates morphology and molecular alterations is extremely important for therapeutic strategies and the prognosis of HCC. In future studies, we will further investigate this aspect.

In conclusion, we propose the first high-accuracy histopathological classification of hepatocellular carcinoma with deep learning technique. The patch screening and dynamic label smoothing strategies are adopted to handle liver histopathological image with noise annotation from the perspective of input and output. The sufficient experiments demonstrate that our two-step method has the ability to reduce the negative effects of noise. Compared to the regular CNN model, our proposed model has a higher pixel-level accuracy, and it also has an advantage in detecting well-differentiated HCC and MVI. Our work might be of reference value for future research exploring digital pathology diagnosis, mainly reflecting in the results of the screening methods, multiple patch scales, and label smoothing. In the future, the proposed model will be able to subdivide the tumor into several categories if there are sufficient and balanced data. Besides that, liver cirrhosis, hepatitis, and other liver tumors can also be trained with our model as long as the training set contains enough samples, and these additional categories will benefit clinical diagnosis from multiple indicators.

## Data Availability Statement

The original contributions presented in the study are included in the article/**Supplementary Material**. Further inquiries can be directed to the corresponding authors.

## Ethics Statement

The studies involving human participants were reviewed and approved by the Ethics Committee of The First Affiliated Hospital, College of Medicine, Zhejiang University. The patients/participants provided their written informed consent to participate in this study. Written informed consent was obtained from the individual(s) for the publication of any potentially identifiable images or data included in this article.

## Author Contributions

JZ, XZ, and SF conceived the study and wrote the manuscript. WL, XL, WZ, and HZ collected and analyzed the clinical and pathological data. XZ and SF scanned the WSIs of H&E-stained HCC pathological slides. XY, ZF, and MS performed the design and training of the deep learning model. XY and XZ analyzed and verified all the data in the study and take responsibility for the integrity of the data and the accuracy of the data analysis. All authors contributed to the article and approved the submitted version.

## Funding

This work was supported by the Natural Science Foundation of Zhejiang Province (LY21H160035), the National Natural Science Foundation of China (81971686).

## Conflict of Interest

The authors declare that the research was conducted in the absence of any commercial or financial relationships that could be construed as a potential conflict of interest.

The editor JC has declared a shared parent affiliation with the authors at the time of review.

## Publisher’s Note

All claims expressed in this article are solely those of the authors and do not necessarily represent those of their affiliated organizations, or those of the publisher, the editors and the reviewers. Any product that may be evaluated in this article, or claim that may be made by its manufacturer, is not guaranteed or endorsed by the publisher.
