# Supplementary material for: Development of a Deep Learning Model to Assist With Diagnosis of Hepatocellular Carcinoma
Source: Front Oncol. 2021 Dec 1;11:762733. doi: 10.3389/fonc.2021.762733 (PMC8671137; doi:10.3389/fonc.2021.762733)
Supplement: Supplementary file 1 [file DataSheet_1.pdf]

Supplementary Figure The visualization results of 20 elaborate annotated slides. There are five slides for each grade.

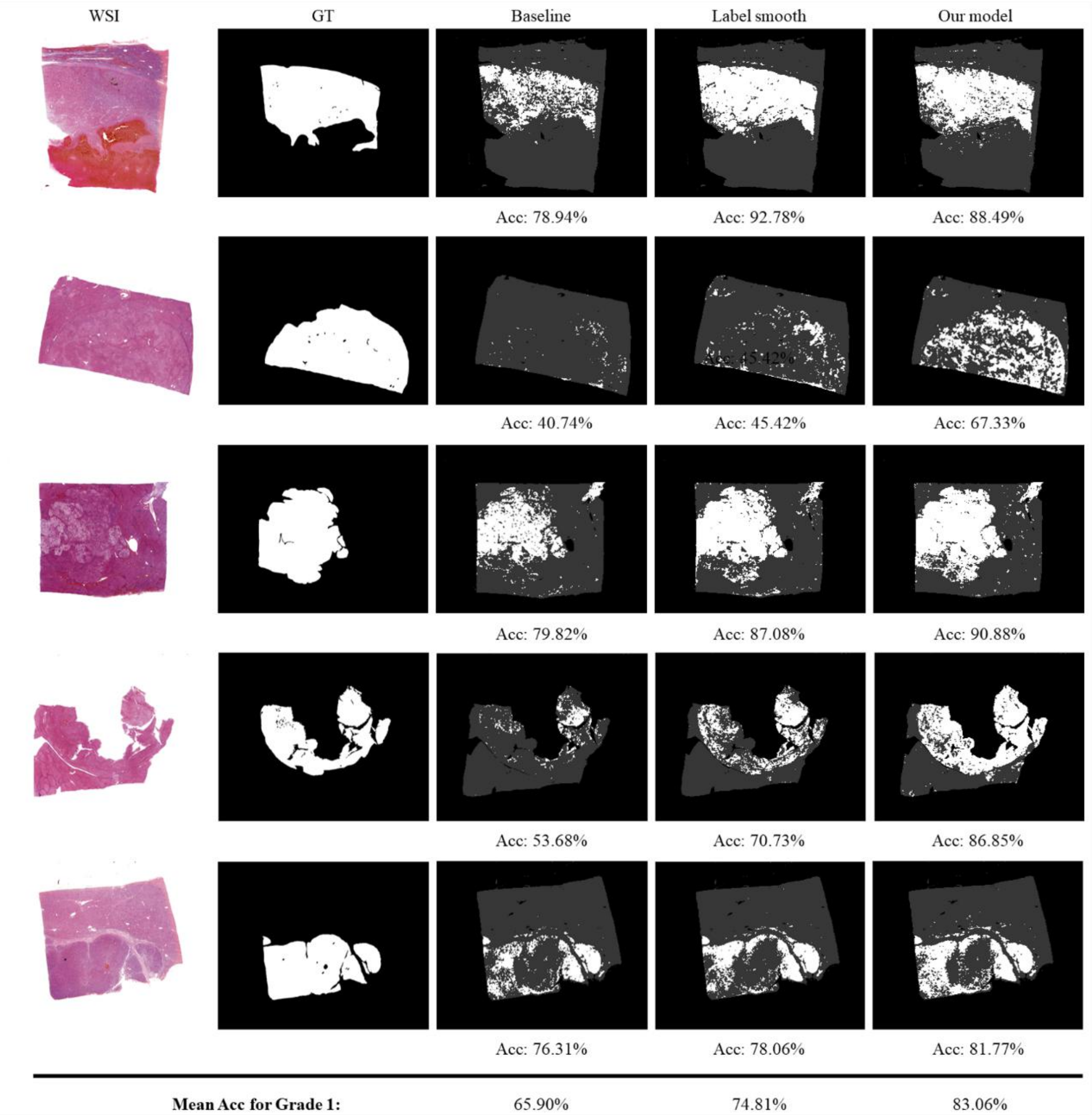

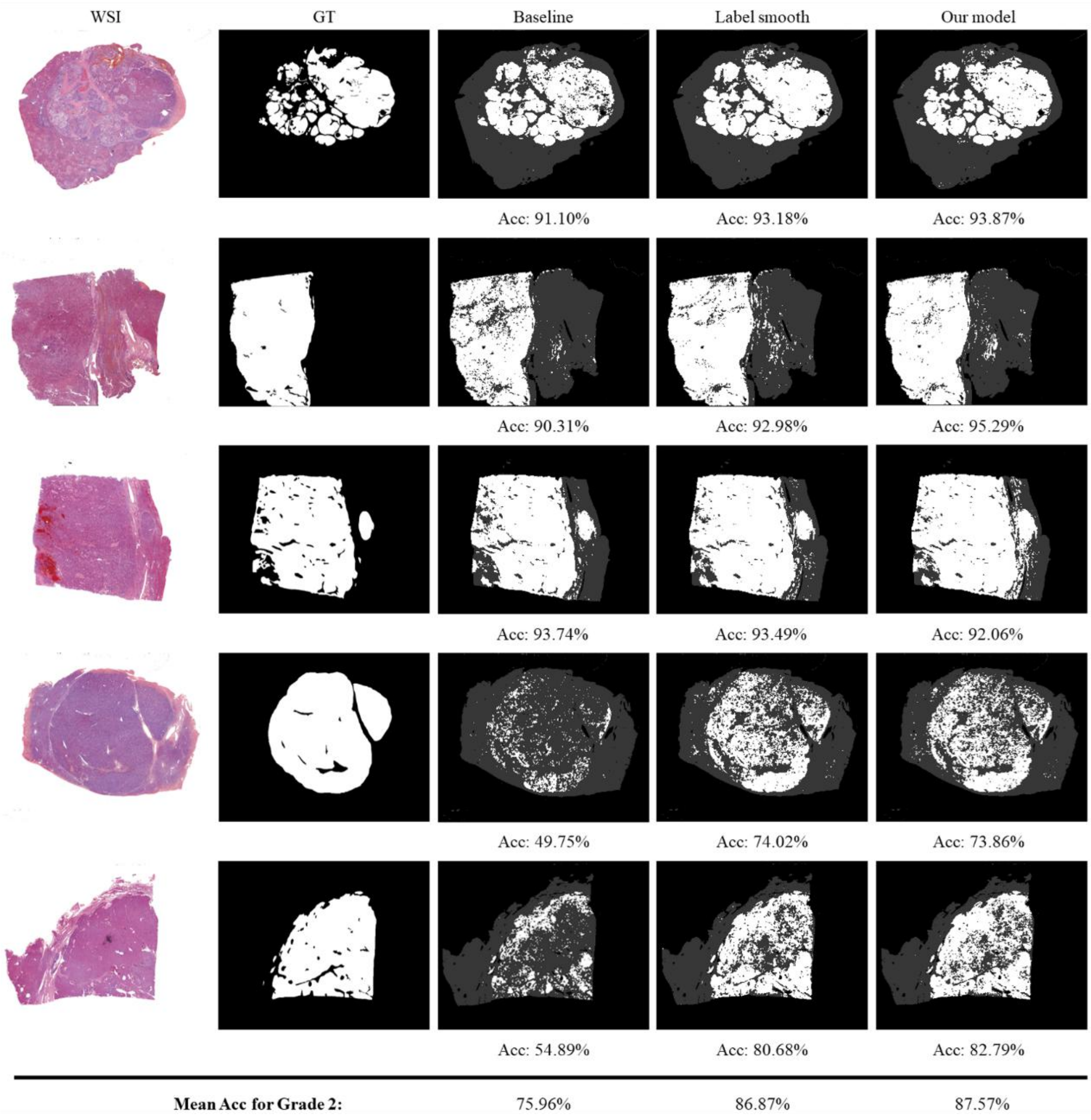
WSI
GT
Baseline
Label smooth
Our model
Acc: 91.10%
Acc: 93.18%
Acc: 93.87%
Acc: 90.31%
Acc: 92.98%
Acc: 95.29%
Acc: 93.74%
Acc: 93.49%
Acc: 92.06%
Acc: 49.75%
Acc: 74.02%
Acc: 73.86%
Acc: 54.89%
Acc: 80.68%
Acc: 82.79%
Mean Acc for Grade 2:
75.96%
86.87%
87.57%

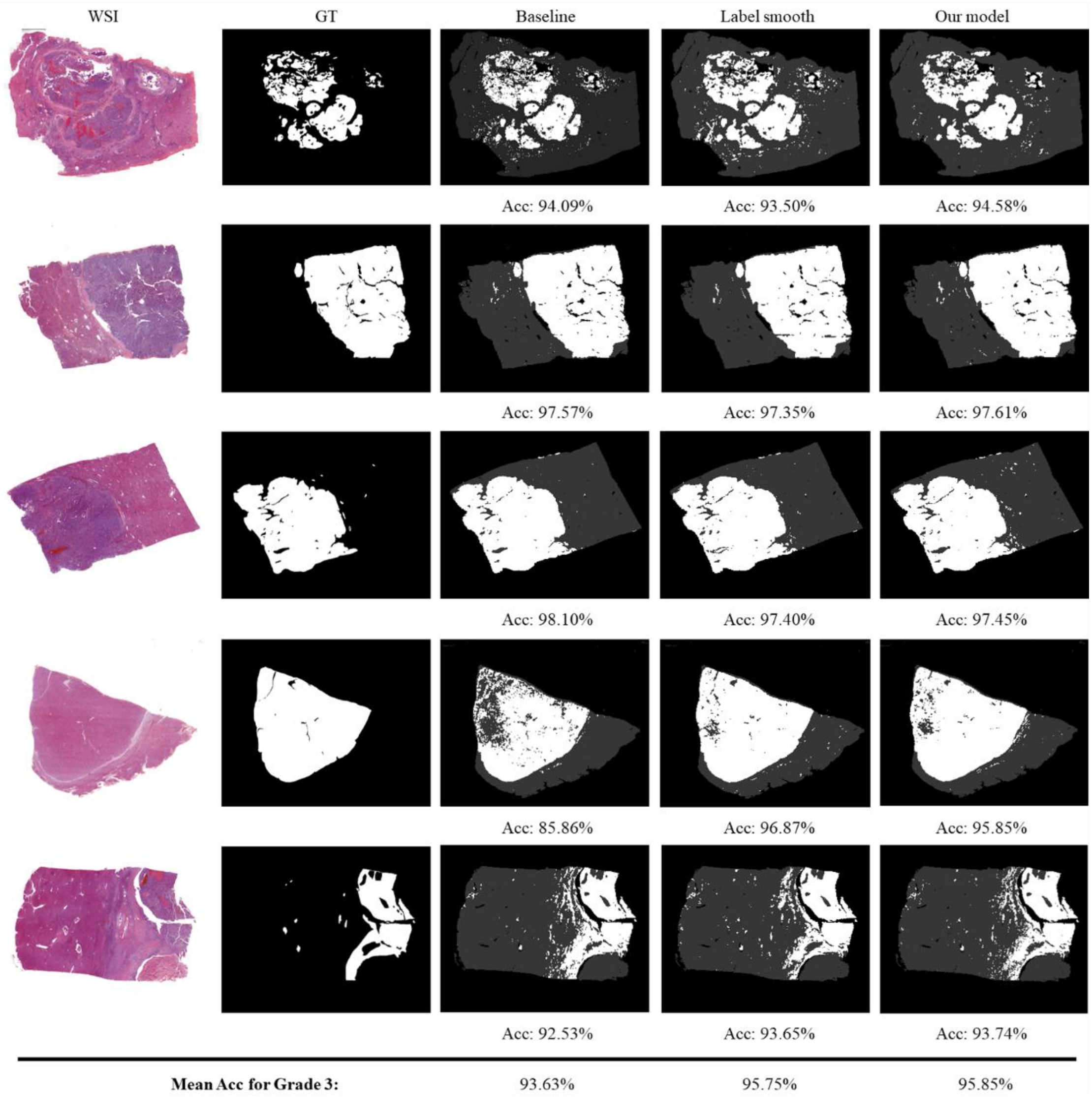
WSI
GT
Baseline
Label smooth
Our model
Acc: 94.09%
Acc: 93.50%
Acc: 94.58%
Acc: 97.57%
Acc: 97.35%
Acc: 97.61%
Acc: 98.10%
Acc: 97.40%
Acc: 97.45%
Acc: 85.86%
Acc: 96.87%
Acc: 95.85%
Acc: 92.53%
Acc: 93.65%
Acc: 93.74%
Mean Acc for Grade 3:
93.63%
95.75%
95.85%

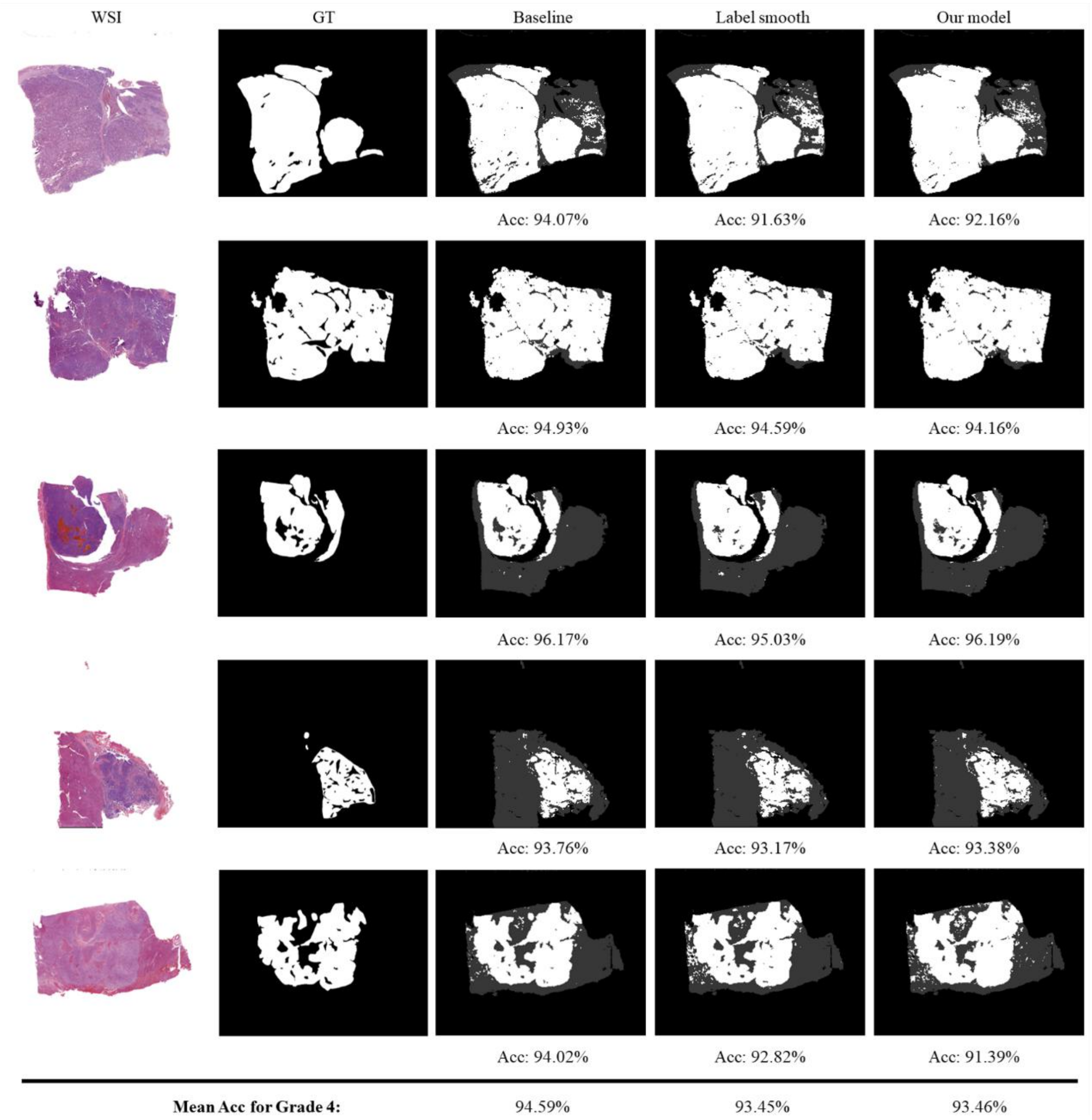

SUPPLEMENTARY MATERIAL

Here we show the visualization results of 20 elaborate annotated slides. There are five slides for each grade so that the model generalization can be better proofed. The overall accuracy of the CNN model with cross-entropy loss (baseline) is 82.52%, while our proposed model achieves 87.81%. Slides of grade 1 have low malignant degrees and are difficult to distinguish. Visualization results show our model is more accurate for these samples. The same conditions are reflected in some slides of grade 2 and grade 3, especially some ambiguous regions in the tumor. As for slides of grade 4, the two models' predicted results are basically the same.
